# Case report: Ultrasound diagnosis of a complicated case of gastric lymphoma misdiagnosed as cirrhosis

**DOI:** 10.3389/fonc.2024.1362555

**Published:** 2024-04-15

**Authors:** Dayan Yang, Lini Gao

**Affiliations:** Department of Ultrasonography, Hainan General Hospital (Hainan Affiliated Hospital of Hainan Medical University), Haikou, Hainan, China

**Keywords:** ultrasound, gastric lymphoma, cirrhosis, diagnosis, CEUS

## Abstract

The gastrointestinal (GI) tract is the most common primary site for extranodal lymphomas. The use of ultrasonography for diagnosing gastric lymphomas can be challenging, but ultrasonography still offers some unique advantages in the diagnosis of GI lymphomas. Here, we report a case of gastric lymphoma in a patient with an extensive lesion in which the tumor was complexed with the abdominal organs. CT and endoscopy failed to definitively diagnose the condition in a timely manner. The gastric lymphoma was finally diagnosed with ultrasonography and a treatment plan was implemented.

## 1 Introduction

Primary gastric lymphomas (PGLs) have low prevalence. Diffuse large B-cell and mucosa-associated lymphoid tissue lymphomas are the two main histologic subtypes of PGLs but have different clinicopathologic features (1). The most common PGL histologic subtype is a primary gastric diffuse large B-cell lymphoma, which most often occurs in males 50-60 years of age. The incidence of PGLs has been increasing worldwide, and some possible risk factors, such as immunosuppression, Helicobacter pylori infection, HIV infection, celiac disease, and EBV, have been associated with the onset of PGLs (2), but non-specific abdominal symptoms (e.g., abdominal discomfort, nausea and vomiting, and weight loss) often lead to a delay in diagnosis. A combination of surgery, chemotherapy, radiotherapy, and antibiotics has been used in the clinical management of PGLs (3). Due to the increasing incidence of PGLs, improving the diagnostic rate can help patients receive treatment in a timely fashion, thereby improving the prognosis and outcomes.

Here, we report a case of gastric lymphoma in a patient with an extensive lesion in which the tumor was complexed with the abdominal organs. CT and endoscopy failed to definitively diagnose the condition in a timely manner. The gastric lymphoma was finally diagnosed with ultrasonography and a treatment plan was implemented.

## 2 Case description

The patient was 82 years of age and had abdominal distension for 40 days without an apparent cause. The abdominal distension was obvious when he was lying down. The abdominal circumference increased progressively, and the abdominal distension and abdominal pain worsened progressively, accompanied by acid reflux and eructation. Anal defecation and a decrease in food intake relieved the abdominal distension. The stools were dry and granular, then pasta-shaped, and became dilute with a soya sauce color. There was no nausea or vomiting. After CT scanning of the entire abdomen, he was diagnosed with cirrhosis, ascites, and peritonitis, and was given an oral diuretic, intestinal flora regulation, and cefmetazole anti-infective treatment. This treatment resulted in a slight improvement in the abdominal distension, but he was later admitted to the hospital with cirrhosis and required emergency treatment. He was hospitalized in the Department of Cardiology of our hospital in April 2014 and diagnosed with coronary artery disease and a non-ST-segment elevation myocardial infarction. He underwent coronary angiography and one stent was implanted in each of the systolic and anterior descending branches. He was prescribed clopidogrel and atorvastatin regularly for secondary prevention of coronary heart disease. He had a history of allergy to sulfonamides, penicillin, streptomycin, and azithromycin, and has smoked approximately 20 cigarettes/day for 20 years.

The vital signs were as follows: temperature, 36.7°C; pulse, 74 beats/min; respirations, 20/min; and blood pressure, 125/51 mmHg. The physical examination was significant for the following: heart rhythm, Qi; no heart murmurs; abdomen, distended; no abdominal wall varicose veins; abdominal muscles, slightly tense; right upper abdomen, pain to superficial pressure and rebound pain; liver and spleen palpation, not satisfactory, The mobile turbidity was positive; bowel sounds 4 times each minute; and mild, pitting edema of both lower extremities.

Upon admission to the hospital, laboratory testing revealed the following: hepatitis E, positive; alpha-fetoprotein, carcinoembryonic antigen, CA-125, and CA19-9, negative; elevated leukocyte count, 11.13×109 per liter; elevated C-reactive protein, 181.78 mg/L; and EBV DNA, negative. The patient improved with treatment for cirrhosis of the liver with ascites.

**Figure 1:**
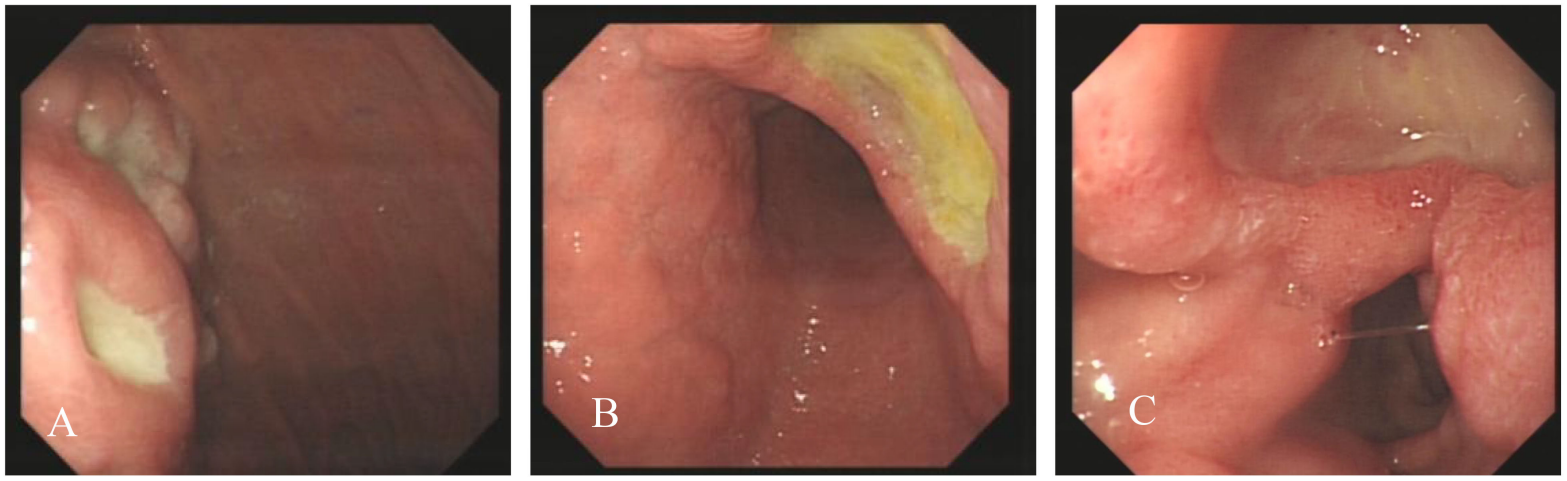
Endoscopic images, Ulcer at the fundus junction of the gastric body **(A)**; ulcers at the small bend of the sinus junction **(B, C)**: ulcers at the descending duodenal bulb **(C)**.

On the first gastroscopy the patient had two 1.0 cm ulcers at the fundus junction of the gastric body (**Figure 1A**), 2.0 cm ulcers at the small bend of the sinus junction (**Figure 1B**), yellow moss, surrounding mucosal congestion and oedema, 1.5 cm ulcers at the descending duodenal bulb (**Figure 1C**) with yellow moss and diverticulum. Multiple ulcers of the gastric duodenum were considered and biopsy was not performed as the patient was on anticoagulant medication.

**Figure 2:**
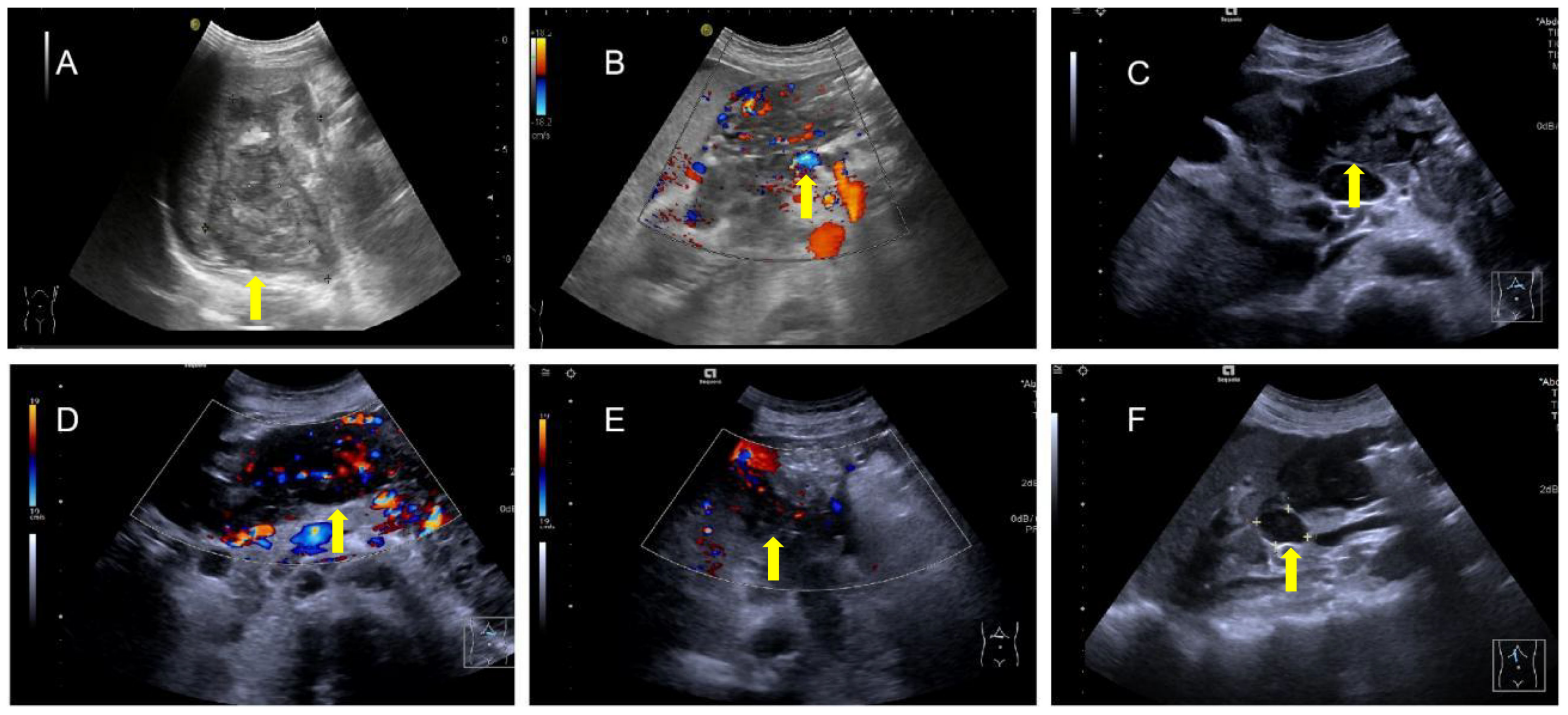
Irregular hypoechoic area in the splenic hilar region **(A)** encompassing the splenic vein **(B)** with several blood flow signals noted in this area and a very hypoechoic area in the left middle abdomen to the splenic hilar region, which is poorly demarcated from the gastric wall, gastric sinus, and spleen **(C)**, and rich blood flow signals in the area **(D)** with thickening of the greater omentum and enhanced echogenicity and several blood flow signals on the omentum, **(E)**, and several lymph nodes in the abdominal cavity and adjacent to the abdominal aorta **(F)**.

**Figure 3:**
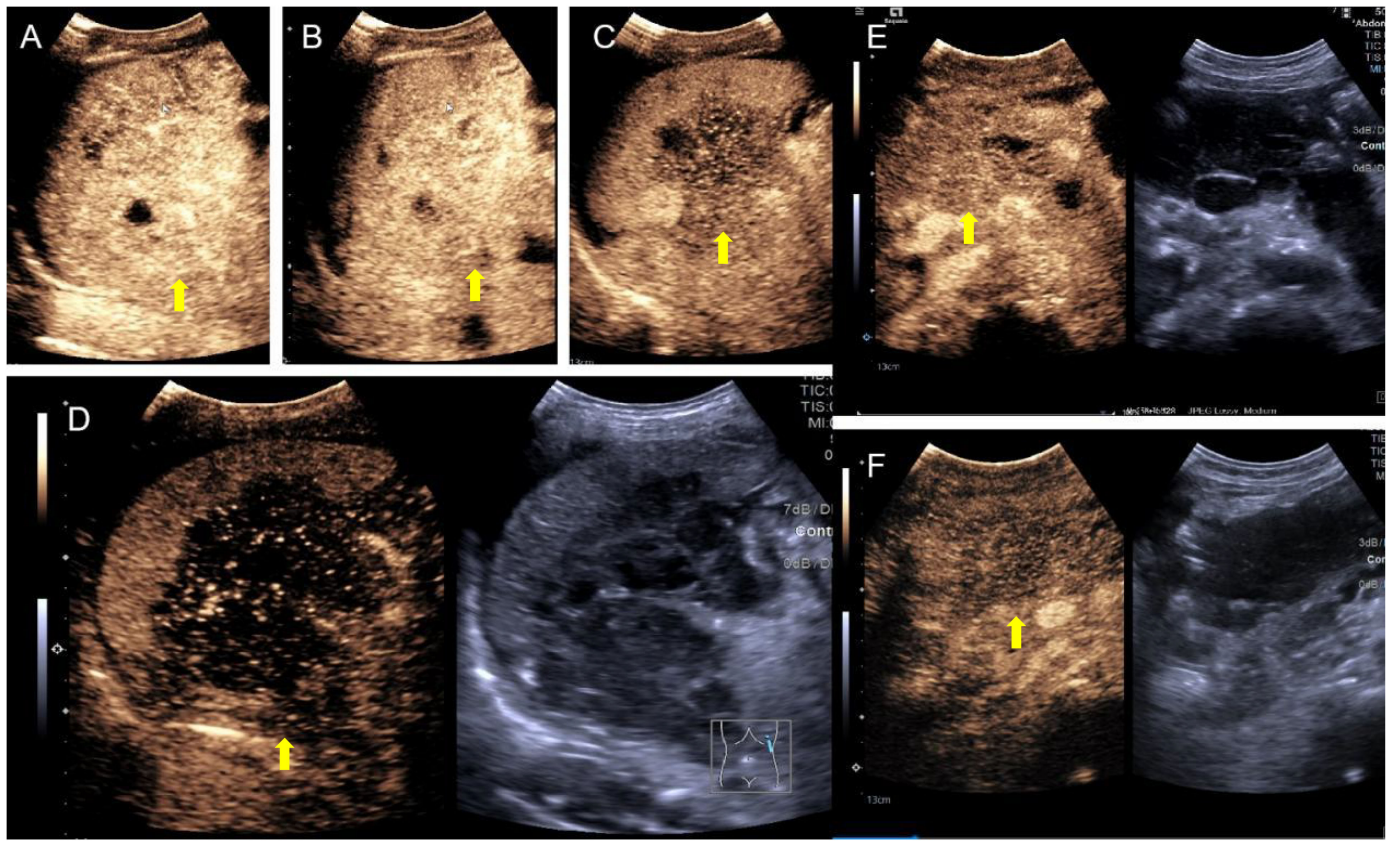
SonoVue contrast-enhanced ultrasound. **(A)** Hyper-enhancement of the mass in the early arterial phase at 13 seconds; **(B)** localized washout at 18 seconds; **(C)** hypo-enhancement area of the mass increased at 1 min and 40 seconds; **(D)** significant washout showed approximately no enhancement at 2 min and 54 seconds; **(E)** iso-enhancement of the abdominal lymph nodes at 1 min and 7 seconds; **(F)** hypo-enhancement of the left mid-abdominal mass at 1 min and 52 seconds.

The patient underwent abdominal ultrasonography, which revealed the following: an irregular hypoechoic area in the splenic hilar region that resembled “coral” and was continuous with a patchy hypoechoic area in the lower part of the spleen; the splenic vein is wrapped around this hypoechoic zone, with no widening; a patchy, very hypoechoic area was noted in the left middle abdomen to the splenic hilar region, spanning 230 × 80 mm and poorly demarcated from the gastric wall, gastric sinus, and spleen with a rich blood flow signal; a thickened (36 mm), echogenic greater omentum; color Doppler flow imaging (CDFI)show a little blood flow signal noted in the omentum; several lymph nodes visualized in the abdominal cavity and adjacent to the abdominal aorta, the larger of which was 25 mm×18 mm; and CDFI, a little blood flow signal was detected in the lymph nodes (**Figures 2A-F**). SonoVue (2.0 ml; Bracco, Milan, Italy) was injected through the median cubital vein, and a lesion from the left upper mid-abdomen to the splenic hilar region was observed with diffuse inhomogeneous hyper-enhancement in the arterial phase, and gradual contouring and very low enhancement in the venous phase. A patchy, very hypoechoic area was noted in the left mid-abdomen to the splenic hilar region, as well as multiple enlarged lymph nodes in the abdominal cavity and retroperitoneum when combined with contrast-enhanced ultrasound (CEUS), suggesting neoplastic lesions (poorly demarcated from the gastric wall and spleen) and lymphomas (**Figures 3A-F**).

**Figure 4:**
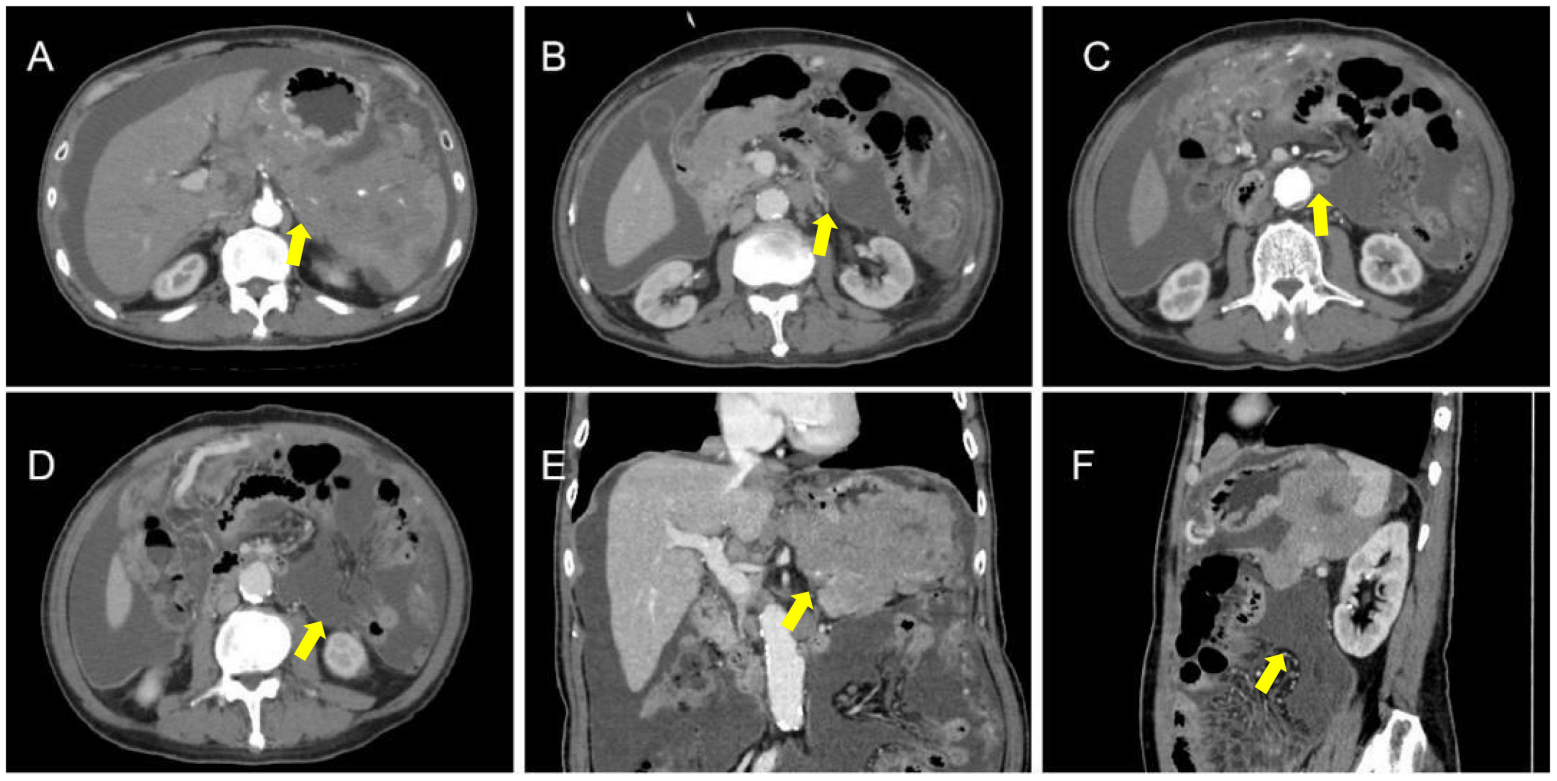
CT apparent irregular thickening of the gastric fundus and sinus wall with enlargement of multiple lymph nodes **(A-D)**, suggesting a neoplastic lesion, such as gastric cancer with lymph node metastases, and localized peritoneal thickening in the form of nodules and masses, suggesting peritoneal metastases **(E, F)**.

**Figure 5:**
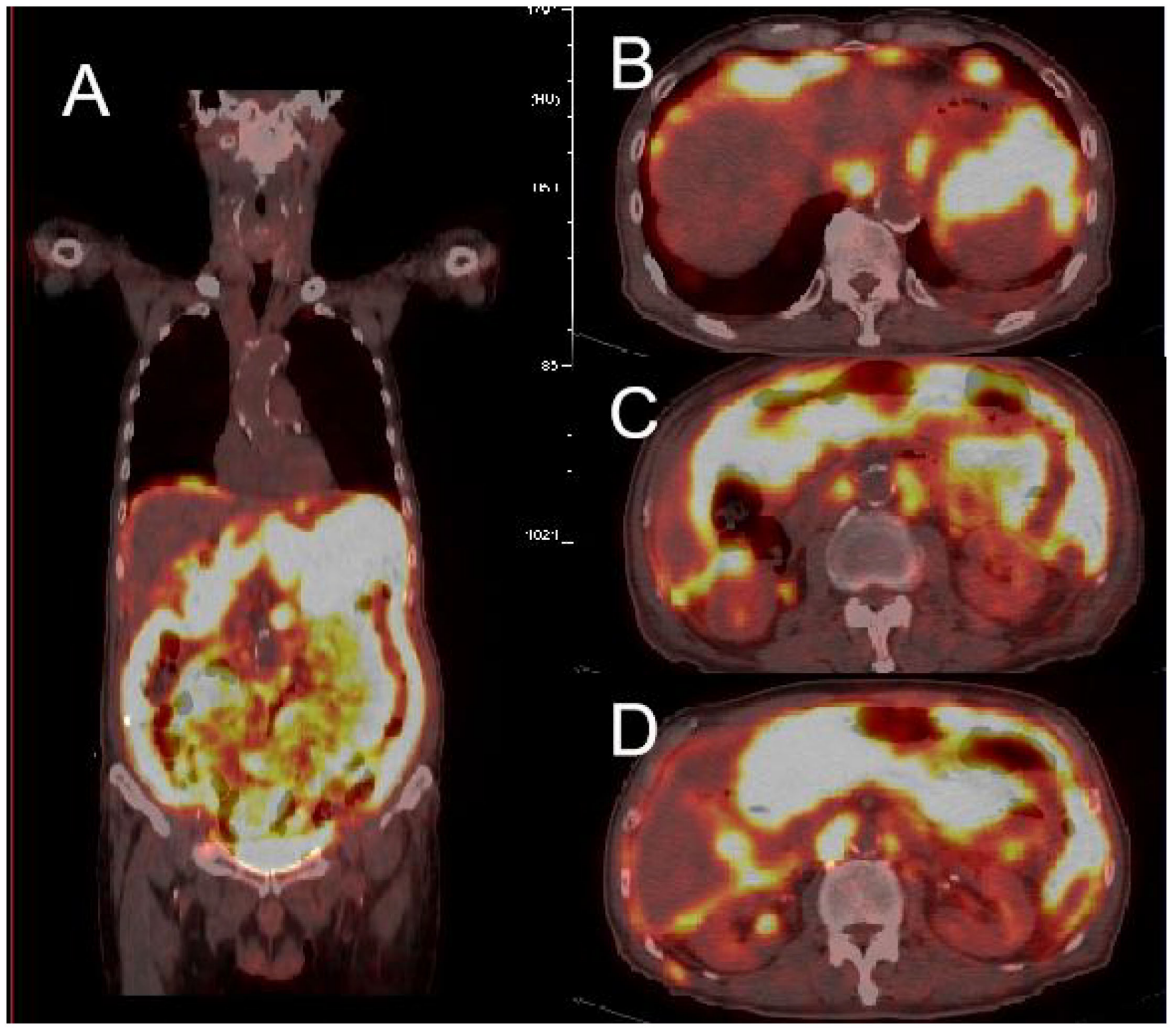
PET-CT showed high uptake of 18F-FDG in multiple abdominal organs **(A)**, with the larger uptake located in the splenogastric space and poorly demarcated from the spleen and stomach **(B-D)**.

The patient underwent abdominal CT enhancement examination, which revealed irregular thickening of the gastric fundus and gastric sinus wall with enlargement of multiple lymph nodes, consistent with a neoplastic lesion and gastric cancer with lymph node metastases. The localized peritoneal nodular and mass thickening suggested peritoneal metastases. A large volume of ascites and peritonitis was noted (**Figure 4A-F**). A PET-CT (the co-expression agent was 18F-FDG) showed the following: 1. enlargement of multiple lymph nodes in the mediastinum, left medial breast, and cardiophrenic angle area, with increased FDG metabolism; 2. multiple nodules and masses in the abdominal cavity, and larger nodules and masses are located in the splenic-gastric hiatus and are poorly delineated from the spleen and stomach with increased FDG metabolism; 3. thickening of the hepatic and splenic peri-membranes with increased FDG metabolism; and 4. diffuse thickening of the peritoneum and mesentery with FDG metabolism, suggesting a malignant neoplastic lesion, such as a lymphoma or sarcoma (**Figure 5**).

**Figure 6:**
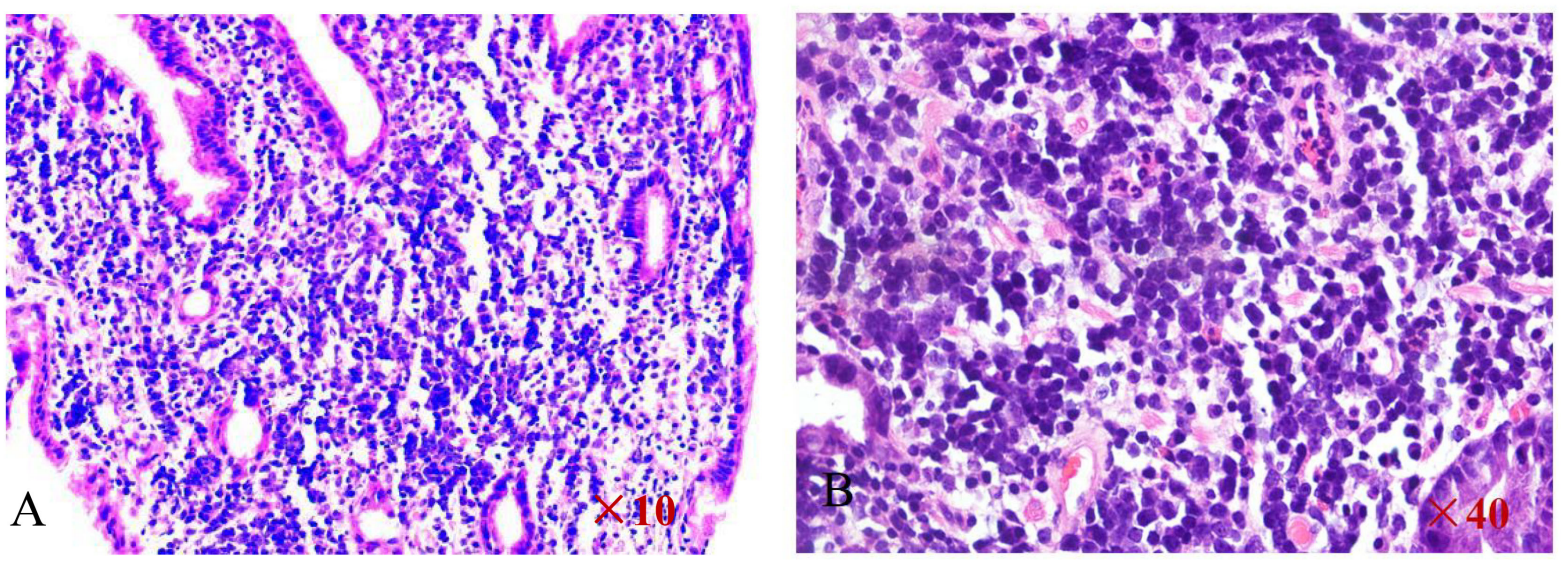
HE staining of the pathological section reveals diffuse enlargement of large B-cell lymphocytes **(A, B)**.

Because all of the above examinations were considered to represent neoplastic lesions of the stomach, the patient underwent a second gastroscopy to confirm the diagnosis and tissues were obtained for pathologic examination. 4 grains of grey - white tissue sent for examination. The pathological section revealed diffuse enlargement of lymphocytes, nuclear divisions are more visible(**Figures 6A, B**). The immunohistochemical results of the gastric antrum were as follows:CD20 (B cell diffuse +), PAX-5 (B cell diffuse +), CyclinD1(-), CD3(-),CD23(-),CD21(-),CD5(-),CD10(-),Bcl-2(+,about 90%),Bcl-6(+,about 30%),C-Myc(+,about30%),WUW1(+),P53(+),CD79a(B cell diffuse +),Ki-67(+,about 80%),CD30(-),CD19(B cell diffuse +).Results of the molecular pathology examination: EBER-EBER(-). Combination with the immunohistochemical findings, the final diagnosis was gastric diffuse large B-cell lymphoma [NOS, COO typing: no-GCB (Hans method)].

After a series of examinations, the diagnosis of a lymphoma was clear. Considering the patient’s age and underlying diseases, it was thought that chemotherapy would be poorly tolerated. Therefore, palliative care was considered a better treatment option. Elderly patients can benefit from chemotherapy, but there are numerous adverse reactions and a high mortality rate. The patient and his family members expressed their understanding of the risks and benefits and agreed to chemotherapy. The patient had cardiac disease, so anthracyclines are avoided. VP16 was given instead of an anthracycline. Combination therapy with R-COEP and nutritional supportive therapy. The patient developed myelosuppression after chemotherapy. The patient was older and had underlying diseases. He developed infectious shock, respiratory failure, and circulatory failure. Resuscitative efforts were ineffective and the patient died.

## 3 Discussion

The current case was a complicated consultation. The emergency CT did not identify a gastric mass. The physical examination revealed progressive enlargement of the abdominal circumference and abdominal distension, which was consistent with the abdominal pain. Treatment targeted the cirrhosis, ascites, and peritonitis. Although there was symptomatic improvement, the therapeutic effect was not good. To escalate treatment, the patient was admitted to the hospital. The patient was infected with hepatitis E. Laboratory testing showed inflammation and negative indicators of gastrointestinal tumors. Gastroscopy did not reveal any masses, which further hindered the diagnosis; however, the final imaging tests, especially ultrasonography, were very helpful in diagnosing a tumor. Based on the morphology of the mass and the mode of growth, a lymphoma was considered and the diagnosis was finally confirmed. Ultrasound had a unique role in the relevant investigations for this patient and had a key role in confirming the diagnosis of a gastric lymphoma. According to the CT enhancement report, a space-occupying lesion was detected, which was considered a gastric carcinoma, even though gastroscopy did not confirm the space-occupying lesion. Previous studies have shown that a CT examination of the upper abdomen is the most accurate imaging examination in the diagnosis of PGLs. In this case the misdiagnosis of gastric cancer may be due to the fact that CT generates a static set of images that visualize space occupying lesions without an impact on gastric function. Whether the mass was soft or rigid could not be determined. Ultrasound not only demonstrated the space-occupying lesion, but also showed that gastric peristalsis was not affected, so the tumor was thought to be soft. A gastric lymphoma was distinguished from gastric cancer based on the peristalsis activity and characteristics of the gastric wall. Gastroscopy can only visualize the mucosal layer and a lesion that protrudes into the lumen of the tube. Therefore, the lymphoma diagnosis was missed in this case. The advantages of ultrasound in the differential diagnosis of gastrointestinal tumors were apparent in the current case. When a space occupying lesion was demonstrated on ultrasound, CEUS was performed in time. The arterial phase was shown to be inhomogeneous and hyper-enhanced, the contrast agent start to wash out on early venous phase, Late vein showed significant hypo-enhanced. This performance in CEUS suggested a malignant lesion, which increased the sonographer’s confidence in the diagnosis of a lymphoma.

Primary gastric lymphoma is a malignant lymphoma primary to the lymphoid tissue of the lamina propria of the gastric mucosa. The patient had a wide lesion range and an unclear demarcation between the spleen and the stomach, Therefore, the lesion was easy to confuse with gastric cancer or other malignant neoplastic lesions in the abdominal cavity with metastases. The early stage of a primary lymphoma gastric lesion mainly infringes on lymphoid tissues in the submucosal layer and the muscularis mucosae, and does not usually involve the mucous membrane or the plasma membrane layer. Thickening of the surface of the gastric wall was thread-like. The echogenic bands were not significantly disrupted and interrupted, which becomes a point of differentiation when compared with ultrasound images of gastric adenocarcinoma (4). Gastric cancer and gastric lymphomas have different treatment modalities. PGLs have a strong sensitivity to chemotherapy and significantly more effective than gastric cancer. Therefore, it is necessary to strengthen our understanding of the diagnostic techniques for PGLs. Accurate diagnosis and selection of appropriate chemotherapy regimens based on pathologic immunohistochemistry results are of great benefit to patients.

**Figure 7:**
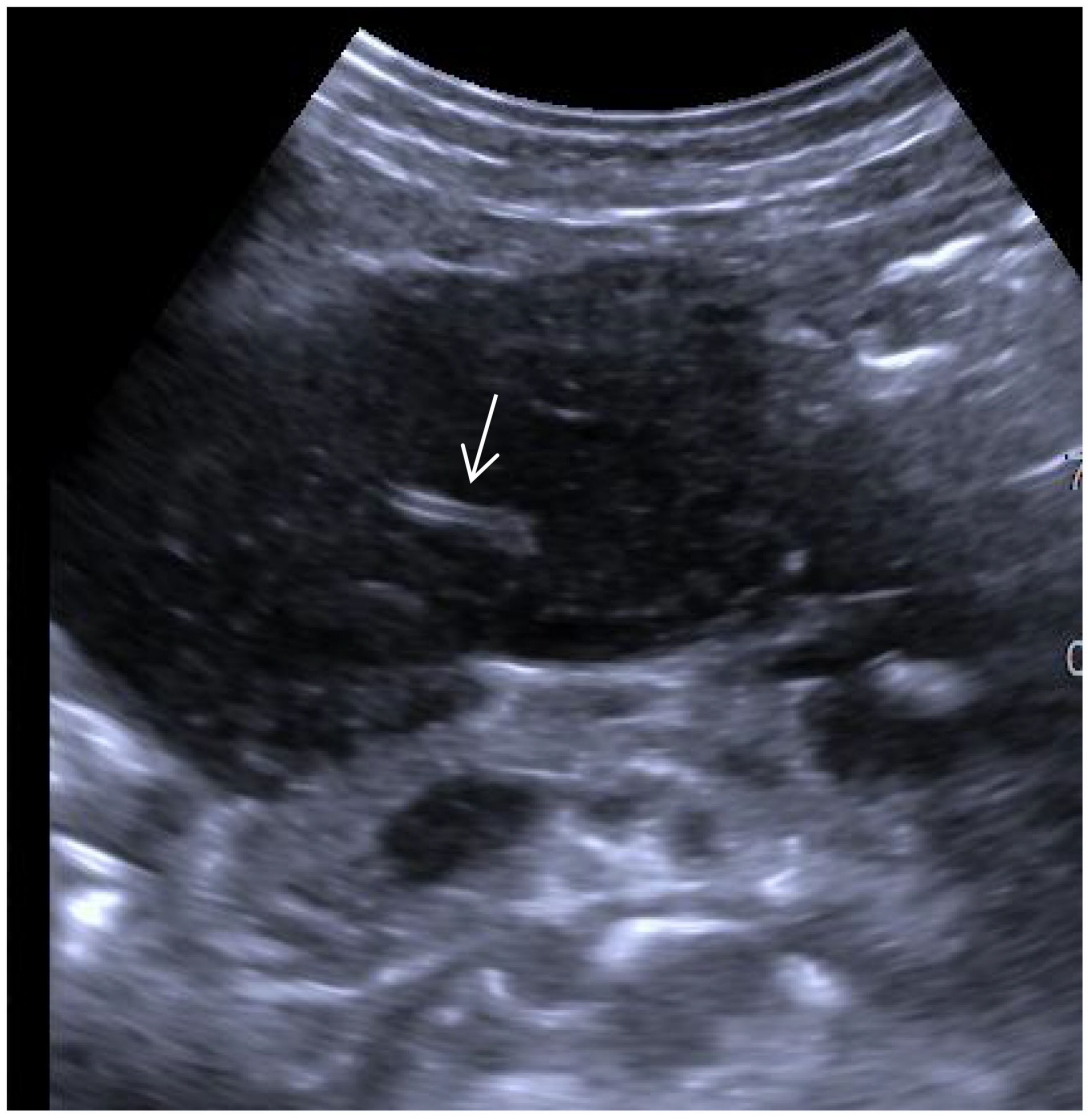
Ultrasound visualization of the vascular floating sign (white arrow).

Ultrasonography has an irreplaceable role in evaluating space occupying lesions in the gastrointestinal tract. It has been suggested that the specificity of conventional ultrasound in the diagnosis of parenchymal extranodal lymphoma is characterized by extreme hypoechoicity (approximate absence of echogenicity), latticework, and the “vascular floatation sign” (5). A vascular floating sign was seen on ultrasonography in this patient (**Figure 7**). The ultrasound manifestations of gastric malignant lymphomas and gastric cancer are similar, and it is difficult to distinguish between them exclusively from ultrasound findings. Early ultrasonographic features include the following: the gastric wall was mildly thickened; low echogenicity; mucosal breakdown; and multiple small shallow mucosal depressions on the surface with a diameter < 5 mm. It is not easy to distinguish erosive gastritis from early gastric cancer. The ultrasound characteristics of PGLs are as follows: thickening of the gastric wall is large (≥50 mm); the thickness is relatively thin (20 mm); thickening of the gastric wall or the interior of the mass is hypoechoic or nearly echo-less, and the sound transmission is excellent; high-frequency ultrasonography can show nodular changes in some of the masses and the gastric lumen is not seriously obstructed and the rigidity of the gastric wall is not apparent. Sonographic typing: ulcer, mass, and diffuse infiltration types, with the ulcer type being the most common. Endoscopic ultrasonography can see the tumor more clearly and distinguish its fine structure, but transabdominal ultrasonography is easy to operate, less painful for patients, and convenient for patients to follow up and observe. Combined with contrast ultrasound technology, the diagnosis rate of gastric lymphoma can be improved, which can be used as the primary choice for routine screening and referral. Contrast ultrasound technology can help us to differentiate the benign and malignant of the space, and can clarify the boundary of the mass. The contrast agent used by contrast ultrasound imaging technology is safe, fast metabolism, does not cause the burden of liver and kidney, and uses a wide range of people.

The shortcoming of this case was that the patient did not undergo an ultrasound gastrointestinal filling contrast examination, and observations of the layers, functions, and mucosa of the gastric wall at the tumor site were lacking. In our future work, we should apply ultrasound of the gastrointestinal tract to provide more reliable diagnostic information and help patients with a clear diagnosis.

## Data availability statement

The original contributions presented in the study are included in the article/supplementary material. Further inquiries can be directed to the corresponding author.

## Ethics statement

The studies involving humans were approved by Department of Ultrasonography, Hainan General Hospital (Hainan Affiliated Hospital of Hainan Medical University). The studies were conducted in accordance with the local legislation and institutional requirements. The participants provided their written informed consent to participate in this study. Written informed consent was obtained from the individual(s) for the publication of any potentially identifiable images or data included in this article. Written informed consent was obtained from the patient for the publication of this case report.

## Author contributions

DY: Writing – original draft, Writing – review & editing. LG: Data curation, Resources, Supervision, Writing – original draft, Writing – review & editing.
